# Is Speech the New Blood? Recent Progress in AI-Based Disease Detection From Audio in a Nutshell

**DOI:** 10.3389/fdgth.2022.886615

**Published:** 2022-05-16

**Authors:** Manuel Milling, Florian B. Pokorny, Katrin D. Bartl-Pokorny, Björn W. Schuller

**Affiliations:** ^1^EIHW–Chair of Embedded Intelligence for Health Care and Wellbeing, University of Augsburg, Augsburg, Germany; ^2^Research Unit iDN–interdisciplinary Developmental Neuroscience, Division of Phoniatrics, Medical University of Graz, Graz, Austria; ^3^Division of Physiology, Otto Loewi Research Center, Medical University of Graz, Graz, Austria; ^4^GLAM–Group on Language, Audio, & Music, Imperial College London, London, United Kingdom

**Keywords:** artificial intelligence, disease detection, healthcare, machine learning, speech

## Abstract

In recent years, advancements in the field of artificial intelligence (AI) have impacted several areas of research and application. Besides more prominent examples like self-driving cars or media consumption algorithms, AI-based systems have further started to gain more and more popularity in the health care sector, however whilst being restrained by high requirements for accuracy, robustness, and explainability. Health-oriented AI research as a sub-field of digital health investigates a plethora of human-centered modalities. In this article, we address recent advances in the so far understudied but highly promising audio domain with a particular focus on speech data and present corresponding state-of-the-art technologies. Moreover, we give an excerpt of recent studies on the automatic audio-based detection of diseases ranging from acute and chronic respiratory diseases via psychiatric disorders to developmental disorders and neurodegenerative disorders. Our selection of presented literature shows that the recent success of deep learning methods in other fields of AI also more and more translates to the field of digital health, albeit expert-designed feature extractors and classical ML methodologies are still prominently used. Limiting factors, especially for speech-based disease detection systems, are related to the amount and diversity of available data, e. g., the number of patients and healthy controls as well as the underlying distribution of age, languages, and cultures. Finally, we contextualize and outline application scenarios of speech-based disease detection systems as supportive tools for health-care professionals under ethical consideration of privacy protection and faulty prediction.

## 1. Introduction

The world is continually changing driven by technological progress. By now, there are more mobile phones on our planet than human beings, we are using virtual agents for navigation purposes or to manage our shopping lists, we are searching for partners via digital dating services, and prefer consulting “Dr. Internet” on perceived symptoms over visiting a medical practice. Worldwide, the technological advancements of the recent years have promoted interconnectivity between human beings and machines. In particular, the COVID-19 pandemic starting in 2019 has shown that communication is not restricted to geographic bounds, as we quickly and successfully switched from personal interactions at the same table to virtual interactions from our individual home offices. The health sector was affected by achievements in digital signal transmission and information technology as well. In times in which humans have a longer life expectancy, but partly tend to have an unhealthier lifestyle than ever before, developments in health technology have made it possible that the acquisition of medically relevant parameters does neither necessarily require healthcare professionals, nor an examination room. Today, healthcare monitoring can be done basically everywhere and anytime (1).

More and more people equip themselves with commercial wearables, such as wristbands with integrated sensor technology, to continuously monitor vital parameters and infer activity patterns or fitness status over time. But also in medical science, the collection of health-related data by means of wearable devices has become increasingly popular. Today's sensors have small size, light weight, and long battery life—characteristics that allow high flexibility for data collection in everyday life settings. Such a long-term wearable-based remote tracking of vital signs is well-suited for different application scenarios, such as disease prevention, disease detection, or intervention planning and control. The most frequently used vital parameters tracked by means of wearable devices for medical applications include heart rate, blood pressure, respiration rate, blood oxygen saturation, and body temperature (2, 3). Apart from the direct measurement of vital signs, other data logs such as GPS or acceleration information can be used to indirectly deduce behavioral patterns associated with physical and mental health. Nowadays, appropriate sensors are integrated in all common smartphones. This also holds true for microphones, which are well-suited for recording another type of data with high relevance in the medical domain—speech^1^ data.

Large quantities of data collected in people's natural environment enable the development of novel approaches that have the potential to revolutionize the healthcare system. For the analysis of such data sets for complex patterns and relationships, the use of artificial intelligence (AI) has almost become indispensable today. Even though a number of questions, e. g., regarding ethics or practical implementation, are still open, the starting shot for an era in which diseases are automatically detected by machines to support medical doctors in diagnostic procedures was fired (4). In the following, we aim to address recent advances in the audio domain with a particular focus on speech data and present corresponding state-of-the-art AI technologies. This mini review shall give an excerpt of recent studies on the automatic audio-based disease detection, covering a variety of medical conditions.

## 2. Artificial Intelligence

AI nowadays usually refers to technologies, which are able to solve complex tasks, including pattern recognition or creative tasks, which were previously expected to be only solvable by humans. With advances in AI however, more and more problems appear increasingly easy to solve, thereby further shifting the line of what problems need “true” intelligence to be solved.

Most breakthroughs in recent decades of AI research came from the field of machine learning (ML). ML subsumes several techniques, in which the algorithm designer only provides a learning framework, based on which the algorithm can learn from training data how to make decisions. Among the subfields of ML, supervised learning most importantly contributes to current automatic disease prediction systems. In this approach, each data point is accompanied by a label indicating the target of the ML algorithm. Successful algorithms in this field mostly belong to the group of parametric ML algorithms, which rely on fixed-sized sets of usually continuous-valued parameters used for decision making. The search for a well-suited parameter set is in general realized by an optimization algorithm as a part of the training routine. The target of the optimisation process is to achieve high performance on target evaluation metrics, which depend on the problem and can be categorized in two groups. For regression tasks, i.e., the prediction of a single continuous value, evaluation metrics such as the root mean square error (RMSE) or the concordance correlation coefficient (CCC) are based on the absolute difference of prediction and label for given data points. For classification tasks, i.e., the assignment of a data point to one of different pre-defined classes, common evaluation metrics such as the unweighted average recall (UAR) or accuracy (Acc) are based on the confusion matrix, which displays the relationship of class predictions and class labels.

For most supervised ML tasks the general processing framework is similar: Provided data, often in form of pre-processed features (see Section 3), is fed into an ML algorithm, which is then optimized to achieve a high performance for a defined regression or classification metric. Whilst details on data, pre-processing, ML algorithm and evaluation metrics may differ from case to case, this common concept has seen tremendous success for a plethora of applications (5).

The currently most successful technique for many ML tasks such as self-driving cars or text generation is deep learning (DL), which is based on artificial neural networks (ANNs) building hierarchical structures of neurons and propagating information via matrix multiplications and non-linear functions and is described in more detail in Goodfellow et al. (6). Based on their architecture, ANNs can be divided into different classes. Feed-forward neural networks (FFNNs) consist of a set of fully-connected or dense layers, i.e., each pair of neurons from consecutive layers has an individual weighted connection. In convolutional neural networks (CNNs), consecutive layers are connected via a convolution operation with weight filters, which are applied similarly as in traditional image processing and share parameters across dimensions. ANNs can be further classified according to the performed task. In a generative adversarial network (GAN), for example, two neural networks compete against each other with one trying to create authentic artificial data samples from noise, while the other trying to discriminate between fake samples created by the first neural network and real samples coming from the database (7).

Especially in the context of small data sets, in which ANNs can fail to generalize well from training to test data, more traditional ML algorithms remain quite popular. Particularly for speech-based disease detection, data sets often only contain a few hours of speech, compared to corpora, for instance, for automatic speech recognition (ASR) with approximately several 1,000 h of speech (8). This apparent gap of dominance for deep learning-based approaches in health-related speech tasks has already been pointed out by Cummins et al. (9). A popular approach is the support vector machine (SVM) for classification tasks and the support vector regression (SVR) for regression tasks, respectively. Both approaches are based on the (non-linear) transformation of the input features into a higher-dimensional space, where the data points can be separated by hyperplanes. In contrast, pure statistic-based analyses, for instance related to mean and standard-deviations of features, are in general not counted as AI methods.

## 3. Speech Modality

AI algorithms rely on the processing of signals, which encode relevant information about the task at hand. Signals can thereby be of different nature and, for instance, encode visual or auditory information. Whilst some areas of AI research, including image-based approaches, have obtained a large amount of attention over the years and resulting algorithms are increasingly accepted and focused on in medical research (10, 11), health-related AI research based on audio data is yet limited. Existing audio approaches often focus on speech data, as human speech production requires an interplay of complex anatomical structures and neurological control, encoding both linguistic information (speech content) and acoustic information (speech quality). The audio signal produced by the human speech apparatus can, thus, be potentially influenced on different levels by a multitude of environmental and internal factors including diseases ranging from a simple cold to a neurological disorder.

The raw form in which audio data can be used for subsequent digital processing and analysis is a time- and value-quantised one-dimensional signal originally based on continuous measurements of air pressure waves through a microphone. Even though some ML algorithms, so-called end-to-end systems, are designed to directly process these raw analog-to-digital converted signals, most approaches still rely on an initial extraction of a set of audio features.

**Figure 1:**
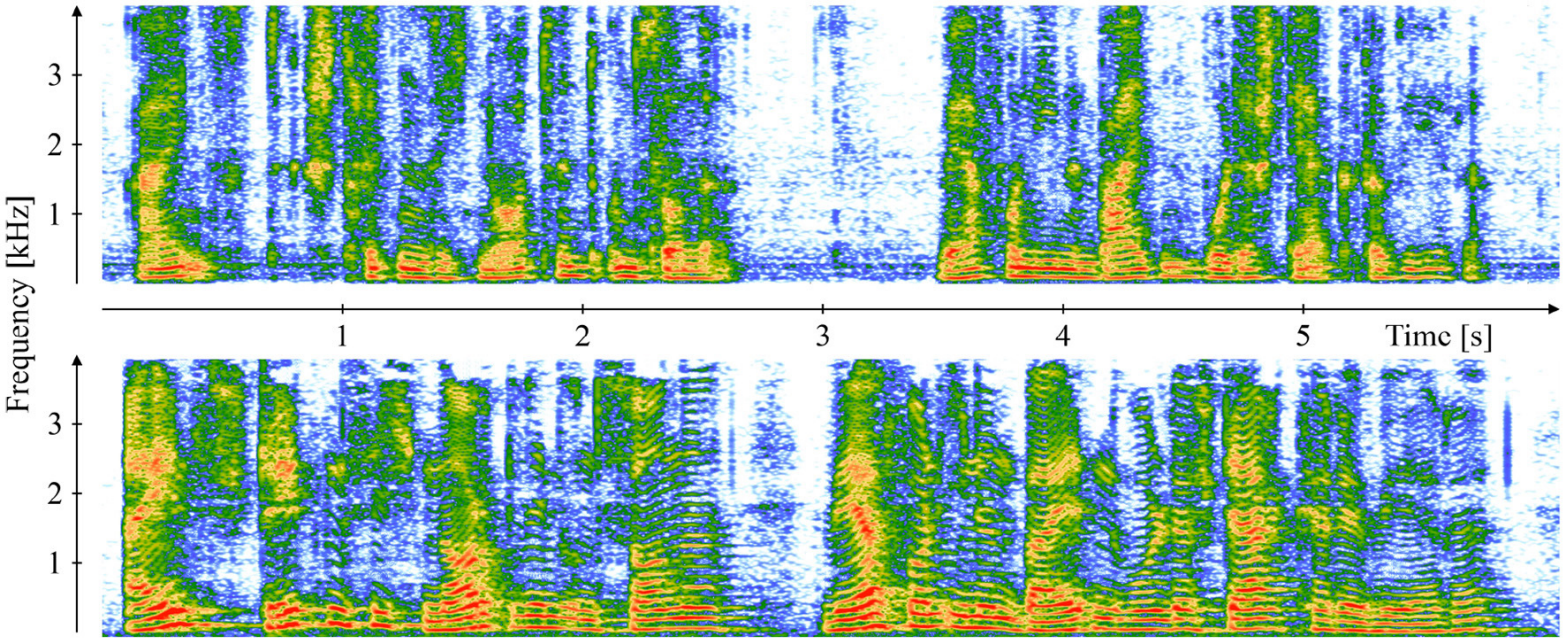
Comparison of a 19-year-old symptomatic male with COVID-19 **(top)** and a 37-year-old asymptomatic COVID-19 negative male **(bottom)** by means of speech spectrograms of the recorded first clause of the German standard text “The Northwind and the Sun” *[“Einst stritten sich Nordwind und Sonne, wer von ihnen beiden wohl der Stärkere wäre."]*. The recordings are part of the “Your Voice Counts” dataset (12, 13).

Traditional audio feature sets rely on a careful expert-driven selection of features potentially relevant for a specific task based on theoretic reasoning and/or practical experience, and they usually include features derived from audio signal representations in different domains, such as the original time-domain or the frequency/spectral domain obtained through a Fourier transform. Figure 1 exemplarily demonstrates speech spectrograms of a patient with COVID-19 and a healthy control. The comparison reveals obvious differences in the frequency domain, mostly toward an increased amount of coarseness in the speech of the patient with COVID-19 reflected in less distinct harmonic overtone structures. In this case, the spectral audio signal representation seems to be a good basis for the derivation of features suited to make an AI system detect a respiratory disease.

Basic properties of the signal are usually captured on a short-term basis through low-level descriptors (LLDs). Examples are the fundamental frequency (*F*_*o*_), jitter, shimmer (14), or Mel frequency cepstral coefficients (MFCCs), which display the short-term energy spectrum on a Mel scale (15), as well as their first (Δ) and second (Δ2) order derivatives. Then, higher-level descriptors (HLDs) are calculated as statistical functionals over LLD trajectories and, thereby, summarize LLDs over larger segments of time. The extended Geneva Minimalistic Acoustic Parameter Set (eGeMAPS) is a rather small standardized set of overall 88 acoustic HLDs selected by experts in the audio field based on their theoretical and practical relevance for automatic speech analysis tasks including clinical tasks (16). In contrast, the Computational Paralinguistics ChallengE (ComParE) feature set represents one of the most extensive standardized feature sets in the field of automatic speech analysis and was introduced as part of the homonymous, yearly Interspeech challenge (17). It comprises 6 373 acoustic HLDs, which are generated through a brute-force combination of numerous LLDs and statistical functionals.

Recently, a number of feature sets, which are not explicitly dependent on expert knowledge, have attracted an increasing amount of attention. This includes the deep spectrum features, which are based on spectrograms, and utilize hidden layers of CNNs, pre-trained on the ImageNet or other image corpora, for feature extraction (18). After the initial feature extraction stage, some approaches exist, which further process these features. A popular approach in this regard is the use of bag-of-audio-words (BoAW) representations to summarize signal characteristics over time by means of their frequency (19).

## 4. Automatic Speech-Based Disease Detection

A literature search in PubMed, one of the most important reference search engines for articles on life sciences and biomedical topics, revealed 85,012 entries on “*artificial intelligence” OR “machine learning” OR “deep learning”* (= search term 1) for the last 5 years (2017–2021) with more than 40% (37,032) of them having been indexed in 2021. These numbers demonstrate an increasing acceptance of AI technology in the health domain. Thereby, research on automated disease detection based on speech data has gained momentum as well: 5,019 of the overall 85,012 entries deal with *speech OR voice OR language* (= search term connected with a logic AND to search term 1) with again more than 40% (2,038) of them stemming from 2021. A number of recently published studies in this field follow supervised ML approaches based on extracted acoustic features as outlined above. However, the exact task, type and size of the data sets, the selection of features and ML algorithms, as well as the corresponding performances vary greatly among the different studies. An excerpt of studies published since 2017 on automated speech-based disease detection^2^ is given in Table 1, specifying above mentioned details.

**Table 1:** Overview of recent speech-based disease detection studies.

**Disease**	**Reference**	**Cohort (m/f)**	**Data**	**Features**	**ML task**	**Appr**.	**Perform**.
Alzheimer's disease	(20)	78, 50–80 y, w/ and w/o, MMST interval: 0–30	Spontaneous speech	BoAW, ZFF-Signals	Prediction of MMST value	SVR	RMSE: 6.97
					w/ vs w/o (2-class)	E2E CNN	Acc: 0.74
Anxiety disorder	(21)	239 (69/170), 18–68 y (31.5 ± 12.3 y), BAI interval: 0–63	Vocalization exercises	ComParE, eGeMAPS, DS	BAI prediction	SVR	ρ ≤ 0.70
Bipolar disorder	(22)	46 (30/16), 18–60 y YMRS: remission (0–7), hypomania (8–19), mania (20–60)	Audio from structured interview	MFCCs	YMRS (3-class)	DNN	UAR: 0.57
Bronchial asthma	(23)	71 (N/A), w/ and 135 (NA) w/o bronchial asthma, 8 ± N/A y	Sustained vowel /a:/	MFCCs, CQCCs	w/ vs w/o (2-class)	GMM-UBM	Acc: 0.72
COVID-19	(24)	52 (32/20), 63.4 ± 9.9 y Hospitalized w/, 3 severity categories	Speech, 5 sentences	eGeMAPS, ComParE	severity prediciton (3-class)	SVM	UAR ≤ 0.68
	(25)	20 (12/8) w/ 60 (40/20) w/o and healthy	Speech	(Δ/Δ2)-MFCCs, LLDs	w/ vs w/o (2-class)	LSTM-RNN	Acc: 0.88
Depression	(26)	275, PHQ-8 interval: 0–24	Audio from semi-clinical interviews	LLDs, BoAW, DS	PHQ-8 prediction	RNNs	CCC ≤ 0.108, RMSE ≥ 8.19
	(27)	292 (N/A), 18–63 y (31.5 ± 12.3 y), BDI-II interval: 0–63	Audio from HCI scenario	log-Mel-spectrograms	BDI-II prediction	CNN	RMSE: 9.65
	(28)	182 (N/A) w/ or w/o, binary PHQ-8	Speech from clinical interviews	MFCC	depression prediction (2-class)	LSTM-RNN	Acc: 0.763
Develop-mental disorder	(29)	11 children w/ ASD, 10 w/ PDD, 13 w/ SLI, 68 typically developed, 6–18 y	Spontaneous speech	eGeMAPS, ComPARE	Developmental disorder prediction (4-class)	GANs	UAR: 0.47
	(30)	10 infants later diagnosed w/ ASD (5/5), 10 typically developed (5/5), 10 m	Audio from PCI scenario	eGeMAPS	ASD prediction (2-class)	SVM, RNN	Acc: 0.75
Parkinson's disease	(31)	23 (N/A) w/ and 8 (N/A) w/o	Speech sound samples	22 selected acoustic features	w/ vs w/o (2-class)	k-NN, RF, NB, SVM	CR ≤ 85.81%
	(32)	50 w/ (25/25) and 50 w/o (25/25), 31–86 y	Read words/texts, monolog, diadochokinetic exercises	488 articulatory features, 28 phonation features, 103 prosody features, 192 glottal features	w/ vs w/o (2-class)	SVM	Acc ≤ 0.68
						E2E CNN	Acc ≤ 0.69
Pathological speech	(33)	126 (N/A)	speech	Cochleogram, Hilbert Spectrum	w/ vs w/o (2-class)	VGG-16 CNN	Acc: 0.92
Upper respiratory tract infection	(34)	630 (382/248), 12–84 y (29.5 ± 12.1 y), w/ and w/o, WURSS-24 (German version)	spontaneous speech, text reading	ComParE	w/ vs w/o (2-class)	DNNs	UAR: 0.67

*For further details, the reader is referred to the original articles. Acc, accuracy; Appr., approach; ASD, autism spectrum disorder; BAI, Beck anxiety inventory; BDI-II, Beck depression inventory-II; BoAW, Bag-of-Audio-Words; CCC, concordance correlation coefficient; CNN, convolutional neural network; ComParE, computational paralinguistics challenge [representations]; CQCCs, constant-Q cepstral coefficients; CR, classification rate; Δ, first derivative; Δ2, second derivative; DNN, deep neural network; DS, deep spectrum [features]; E2E, end-to-end; eGeMAPS, extended Geneva minimalistic acoustic parameter set; GAN, generative adversarial network; GMM-UBM, Gaussian mixture model-universal background model; HCI, human-computer interaction; k-NN, k-nearest neighbor; LLDs, low level descriptors; LSTM, long short-term memory; MFCCs, Mel frequency cepstral coefficients; MMST, mini-mental-status-test; N/A, not available; NB, naive Bayes; PCI, parent-child interaction; PDD, pervasive developmental disorder; m, months; Perform., performance; PHQ-8, 8-item patient health questionnaire depression scale; RF, random forest; ρ, Spearman's Correlation Coefficient; RMSE, root mean square error; RNN, recurrent neural network; SLI, specific language impairment; SVM, support vector machine; SVR, support vector regressor; UAR, unweighted average recall; w/, with [corresponding disease]; w/o, without [corresponding disease]; y, years; ZFF, zero-frequency filtered*.

The presented overview does not claim for completeness, but is intended to indicate current research trends and to reveal the variety of recently done work on AI-driven speech-based disease detection with regard to the used approaches and the addressed disease types alongside the obtained performances. Following a general trend in AI, the most frequently used ML approaches here are ANNs including CNNs and deep neural networks (DNNs), along with SVM/SVR still playing a substantial role, especially for benchmarking purposes and when having only small- to middle-sized datasets available.

The basic feasibility of speech-based disease detection or disease/symptom severity prediction could already be demonstrated for a wide spectrum of medical conditions ranging from acute or chronic respiratory diseases, such as cold and flu (34), COVID-19 (24), or asthma (23), via psychiatric disorders, such as anxiety disorder (21), bipolar disorder (22), or depression (28), to developmental disorders, such as autism spectrum disorder (30), and neurodegenerative diseases, such as Alzheimer's disease (20) or Parkinson's disease (32). Promising results in most of the presented studies suggest that AI-based speech analysis might really have the potential to make a valuable contribution to future healthcare. Efforts should be made to gradually move this technology from a basic research level to its practical application. To this end, a close collaboration between engineers, healthcare professionals, and patient stakeholders will be essential.

## 5. Discussion

En route to make machines automatically analyse human speech to support medical doctors in diagnostic decision-making, a number of obstacles have yet to be overcome. Among the biggest challenges of speech-based AI systems for disease detection is the acquisition of well-controlled and high-quality data of sufficient quantity to apply state-of-the-art AI approaches such as DNNs. To acquire large amounts of data is especially difficult in rare diseases. Moreover, most studies focus on speakers of the same language—in most cases English—and it remains open whether the results are generalisable to other languages. New innovative solutions for the acquisition of bigger and universally interpretable medically relevant speech data are necessary to enhance the potential of AI approaches for disease detection.

### 5.1. Perspectives

Future application fields of speech-based AI systems for disease detection are manifold: Such systems could be used by healthcare professionals in clinics, by local general practitioners or—in case of pandemics such as the COVID-19 pandemic—in specific test centers. There could be specific examination rooms with optimal acoustic conditions where the patient could be asked to produce, e. g., sustained vowels or to read aloud a certain standardized text. The speech material would be recorded by a microphone and immediately analyzed by the AI system. The results would then be interpreted by the healthcare professional and could be used to discuss potential next diagnostic steps or intervention procedures with the patient.

Such AI-based disease detection technology could be even implemented fully automatically during routine examinations, such as the regular examinations of infants and young children in their first years of life. For this, it would only be necessary to equip the examination room with suitable microphones that are connected with the AI system. In the future, such an approach could allow an earlier detection of various developmental disorders that are associated with deficits in the speech-language domain.

Speech-based AI systems could also be applied “in-the-wild,” i.e., in the natural environment of a person. Specific smartphone apps may allow to record speech sequences and provide immediate feedback on the health status of the speaker. They may even directly contact the speaker's general practitioner if a disease is suspected.

Both possible application scenarios—the data collection in a healthcare department or “in-the-wild”—have their own benefits: The data collection in the healthcare department usually results in data of higher quality because the recording environment is easier to control. The data collection “in-the-wild” allows to capture a probably more reliable picture of the actual health status of a person due to the absence of a healthcare professional, cf. the white-coat effect (35), and the presence of a person's familiar environment. Moreover, an in-the-wild data collection would be suited for an efficient and low-resource continuous and individualized observation of a person's health status. This would allow to immediately detect newly occurring atypicalities in a person's speech characteristics and to initiate timely diagnostic procedures.

### 5.2. Ethical Considerations

Without a doubt, AI applications have a high potential to revolutionize the healthcare system. Still, prior to their actual use for disease detection, certain data protection issues need to be solved and ethical questions need to be discussed thoroughly. For example, we need to decide whether speech recordings need to be stored or whether they can be deleted directly after analysis. In case data are stored, it needs to be discussed who can access these data. We need to think about who is allowed to retrieve the disease detection results gained by an AI system. An important limitation of AI-based disease detection systems is that they are based on probability theory and, therefore, may provide misclassifications. Healthcare professionals need to be aware of this and interpret the results of the AI system in the context of other available clinical information. Another critical ethical aspect in connection with AI-based disease detection is raised by the fact that AI systems, especially high-performing state-of-the-art deep learning models, usually represent black boxes hard to understand for human beings. Thus, the clinician would be forced to make a decision based on a result whose genesis is completely unclear to her or him. Hence, the field of explainable artificial intelligence (XAI) deals with techniques to make ML models better understandable and, thus, generated results better interpretable by human professionals (36). A common approach is to derive a selection of features that leads to the best detection performance (37), or to identify those features that contribute most to the final model output. The knowledge about specific speech features that are most essential for the ML algorithm to differentiate between patients with a certain disease and healthy speakers, could allow the physician to draw conclusions about potential voice-physiological atypicalities associated with the investigated disease (38). Alternatively, sonification represents a recently emerging XAI approach, in which sound is generated to auditorily demonstrate salient facets of learning data or relevant acoustic features to allow human listeners to follow the reasoning of an ML algorithm (39).

## 6. Conclusion

This mini review gave an overview of recent progress in the field of automatic speech-based disease detection and revealed promising results for a wide range of medical conditions. At this point, it is essential to highlight that the future goal of AI systems in healthcare is not to replace medical doctors, but rather to serve as an additional examination instrument that can help them to more efficiently and more reliably detect diseases and plan/validate interventions. Future medical doctors will need to learn how to use such AI systems and how to interpret their generated outputs. The healthcare professional will act an essential interface between the AI system and the patient. For a patient, the personal interaction with a healthcare professional is of utmost importance for his or her wellbeing. Taken together, speech has the potential to become similarly important for disease detection as blood is nowadays. In the upcoming years, it will be possible to detect a growing amount of diseases earlier with the help of speech-based AI systems.

## Author Contributions

MM and KB-P conceptualized the work, reviewed the literature, and drafted the manuscript. FP conceptualized the work, reviewed the literature, drafted the manuscript, and created the figure. BS supervised the overall implementation of this work. All authors revised the manuscript and approved the final version of the manuscript.

## Funding

This work was supported by the Bavarian Ministry of Science and Arts through the ForDigitHealth project, funded by the Bavarian Research Association on Healthy Use of Digital Technologies and Media. It has also received funding from the European Union's Horizon 2020 research and innovation programme under grant agreement no. 826506 (sustAGE).

## Conflict of Interest

The authors declare that the research was conducted in the absence of any commercial or financial relationships that could be construed as a potential conflict of interest.

## Publisher's Note

All claims expressed in this article are solely those of the authors and do not necessarily represent those of their affiliated organizations, or those of the publisher, the editors and the reviewers. Any product that may be evaluated in this article, or claim that may be made by its manufacturer, is not guaranteed or endorsed by the publisher.

## Footnotes

^1^Please note that we collectively use the term “speech” for all sounds produced by the human speech apparatus throughout this paper, even though in some context “voice” would be the more precise term from a linguistic point of view.

^2^Please note that we consistently use “detection” as a medical term throughout this work, even though presented studies actually represent classification or regression tasks from a technical perspective.

## References

[1] Panesar A. Machine Learning and AI for Healthcare. Apress. (2019).

[2] Dias D Paulo Silva Cunha J. Wearable health devices-Vital sign monitoring, systems and technologies. Sensors. (2018) 18:2414. 10.3390/s18082414 30044415 PMC6111409

[3] Sheikh M Qassem M Kyriacou PA. Wearable, environmental, and smartphone-based passive sensing for mental health monitoring. Front Digit Health. (2021) 3:662811. 10.3389/fdgth.2021.662811 34713137 PMC8521964

[4] Qian K Li X Li H Li S Li W Ning Z. Computer audition for healthcare: opportunities and challenges. Front Digit Health. (2020) 2:5. 10.3389/fdgth.2020.00005 34713018 PMC8521830

[5] Janiesch C Zschech P Heinrich K. Machine learning and deep learning. Electron Markets. (2021) 31:685–95. 10.1007/s12525-021-00475-2

[6] Goodfellow I Bengio Y Courville A. Deep Learning. MIT Press. (2016). Available online at: http://www.deeplearningbook.org.

[7] Goodfellow I Pouget-Abadie J Mirza M Xu B Warde-Farley D Ozair S. Generative adversarial nets. In: Ghahramani Z Welling M Cortes C Lawrence N Weinberger KQ, editors. Advances in Neural Information Processing Systems. Vol. 27. Curran Associates, Inc. (2014). Available online at: https://proceedings.neurips.cc/paper/2014/file/5ca3e9b122f61f8f06494c97b1afccf3-Paper.pdf.

[8] Panayotov V Chen G Povey D Khudanpur S. Librispeech: An ASR corpus based on public domain audio books. In: Proceedings IEEE International Conference on Acoustics, Speech, and Signal Processing. South Brisbane, QLD: IEEE (2015). p. 5206–10.

[9] Cummins N Baird A Schuller BW. Speech analysis for health: current state-of-the-art and the increasing impact of deep learning. Methods. (2018) 151:41–54. 10.1016/j.ymeth.2018.07.007 30099083

[10] Bolhasani H Mohseni M Rahmani AM. Deep learning applications for IoT in health care: a systematic review. Inform Med Unlocked. (2021) 23:100550. 10.1016/j.imu.2021.100550

[11] Magalhaes C Mendes J Vardasca R. The role of AI classifiers in skin cancer images. Skin Rese Technol. (2019) 25:750–7. 10.1111/srt.12713 31106913

[12] Bartl-Pokorny KD Pokorny FB Batliner A Amiriparian S Semertzidou A Eyben F. The voice of COVID-19: acoustic correlates of infection in sustained vowels. J Acoust Soc Am. (2021) 149:4377–83. 10.1121/10.0005194 34241490 PMC8269757

[13] Hecker P Pokorny FB Bartl-Pokorny KD Reichel U Ren Z Hantke S. Speaking Corona? Human and machine recognition of COVID-19 from voice. In: Proceedings INTERSPEECH. Brno, Czech Republic: ISCA (2021). p. 701–5.

[14] Eyben F Wöllmer M Schuller B. openSMILE-The munich versatile and fast open-source audio feature extractor. In: Proceedings ACM International Conference on Multimedia. Florence: ACM (2010). p. 1459–62.

[15] Davis S Mermelstein P. Comparison of parametric representations for monosyllabic word recognition in continuously spoken sentences. IEEE Trans Acoust Speech Signal Process. (1980) 28:357–66. 10.1109/TASSP.1980.1163420

[16] Eyben F Scherer KR Schuller BW Sundberg J André E Busso C. The Geneva minimalistic acoustic parameter set (GeMAPS) for voice research and affective computing. IEEE Trans Affect Comput. (2015) 7:190–202. 10.1109/TAFFC.2015.2457417

[17] Schuller B Steidl S Batliner A Vinciarelli A Scherer K Ringeval F. The INTERSPEECH 2013 computational paralinguistics challenge: social signals, conflict, emotion, autism. In: Proceedings INTERSPEECH. Lyon: ISCA (2013). p. 148–52.

[18] Amiriparian S Gerczuk M Ottl S Cummins N Freitag M Pugachevskiy S. Snore sound classification using image-based deep spectrum features. In: Proceedings INTERSPEECH. Stockholm: ISCA (2017). p. 3512–6.

[19] Schmitt M Janott C Pandit V Qian K Heiser C Hemmert W. A bag-of-audio-words approach for snore sounds' excitation localisation. In: ITG Symposium on Speech Communication. (2016).

[20] Cummins N Pan Y Ren Z Fritsch J Nallanthighal V Christensen H. A comparison of acoustic and linguistics methodologies for Alzheimer's dementia recognition. In: Proceedings INTERSPEECH (Shanghai: ISCA). (2020). p. 2182–6.

[21] Baird A Cummins N Schnieder S Krajewski J Schuller B. An evaluation of the effect of anxiety on speech–computational prediction of anxiety from sustained vowels. In: Proceedings INTERSPEECH. Shanghai: ISCA (2020). p. 4951–5.

[22] Ren Z Han J Cummins N Kong Q Plumbley M Schuller B. Multi-instance learning for bipolar disorder diagnosis using weakly labelled speech data. In: Proceedings International Conference on Digital Public Health (Marsaille: ACM). (2019). p. 79–83.

[23] Balamurali BT Hee HI Teoh OH Lee KP Kapoor S Herremans D. Asthmatic versus healthy child classification based on cough and vocalised /a:/ sounds. J Acoust Soc Am. (2020) 148:EL253–9. 10.1121/10.0001933 33003873

[24] Han J Qian K Song M Yang Z Ren Z Liu S. An early study on intelligent analysis of speech under COVID-19: severity, sleep quality, fatigue, and anxiety. arXiv. (2020). 10.48550/arXiv.2005.00096

[25] Hassan A Shahin I Alsabek MB. COVID-19 detection system using recurrent neural networks. In: Proceedings IEEE International Conference on Communications, Computing, Cybersecurity, and Informatics. virtual: IEEE (2020).

[26] Ringeval F Schuller B Valstar M Cummins N Cowie R Tavabi L. AVEC 2019 workshop and challenge: state-of-mind, detecting depression with AI, and cross-cultural affect recognition. In: Proceedings International on Audio/Visual Emotion Challenge and Workshop. Nice: ACM (2019). p. 3–12. 10.1145/3347320.3357688

[27] Zhao Z Li Q Cummins N Liu B Wang H Tao J. Hybrid network feature extraction for depression assessment from speech. In: Proceedings INTERSPEECH. Shanghai: ISCA (2020). p. 4956–60.

[28] Rejaibi E Komaty A Meriaudeau F Agrebi S Othmani A. MFCC-based recurrent neural network for automatic clinical depression recognition and assessment from speech. Biomed Signal Process Control. (2022) 71:103107. 10.1016/j.bspc.2021.103107

[29] Deng J Cummins N Schmitt M Qian K Ringeval F Schuller B. Speech-based diagnosis of autism spectrum condition by generative adversarial network representations. In: Proceedings International Conference on Digital Health. London: ACM (2017). p. 53–7.

[30] Pokorny F Schuller B Marschik P Brueckner R Nyström Cummins N. Earlier identification of children with autism spectrum disorder: an automatic vocalisation-based approach. In: Proceedings INTERSPEECH. Stockholm: ISCA (2017). p. 309–13.

[31] Avuçlu E Elen A. Evaluation of train and test performance of machine learning algorithms and Parkinson diagnosis with statistical measurements. Med Biol Eng Comput. (2020) 58:2775–2788. 10.1007/s11517-020-02260-3 32920727

[32] Narendra NP Schuller B Alku P. The detection of parkinson's disease from speech using voice source information. IEEE/ACM Trans Audio Speech Lang Process. (2021) 29:1925–36. 10.1109/TASLP.2021.307836434799077

[33] Gumelar AB Yuniarno EM Anggraeni W Sugiarto I Mahindara VR Purnomo MH. Enhancing detection of pathological voice disorder based on deep VGG-16 CNN. In: Proceedings International Conference on Biomedical Engineering. virtual: IEEE (2020). p. 28–33.

[34] Albes M Ren Z Schuller BW Cummins N. Squeeze for sneeze: compact neural networks for cold and flu recognition. In: Proceedings INTERSPEECH. Shanghai: ISCA (2020). p. 4546–50.

[35] Ogedegbe G. White-coat effect: unraveling its mechanisms. Am J Hypertens. (2008) 21:135–5. 10.1038/ajh.2007.64 18268486

[36] Tjoa E Guan C. A survey on explainable artificial intelligence (XAI): toward medical XAI. IEEE Trans Neural Netw Learn Syst. (2020) 32:4793–813. 10.1109/TNNLS.2020.3027314 33079674

[37] Alghowinem SM Gedeon T Goecke R Cohn J Parker G. Interpretation of depression detection models via feature selection methods. IEEE Trans Affect Comput. (2020) 10.1109/TAFFC.2020.3035535 PMC10019578 36938342

[38] Ren Z Chang Y Bartl-Pokorny KD Pokorny FB Schuller BW. The acoustic dissection of cough: diving into machine listening-based COVID-19 analysis and detection. medRxiv. (2022). 10.1101/2022.03.01.22271693 PMC9197794 35835648

[39] Schuller BW Virtanen T Riveiro M Rizos G Han J Mesaros A. Towards sonification in multimodal and user-friendly explainable artificial intelligence. In: Proceedings International Conference on Multimodal Interaction (Montreal, QC: ACM). (2021). p. 788–92.

